# What is cancer pain? Investigating attitudes of patients, carers, and health professionals: A cross‐sectional survey

**DOI:** 10.1111/papr.70018

**Published:** 2025-03-06

**Authors:** E. Henriksen, J. Young, C. Power, C. Chan

**Affiliations:** ^1^ Department of Anaesthesia and Pain Peter MacCallum Cancer Centre Melbourne Australia; ^2^ Department of Critical Care University of Melbourne Parkville Australia

**Keywords:** cancer, opioids, pain

## Abstract

**Background:**

Cancer pain is a prevalent and debilitating symptom that impacts quality of life. Pain management remains challenging; however, due to various barriers, including stigma associated with opioid use, ambiguous roles of clinicians, and diverse attitudes toward pain management among healthcare professionals, patients, and carers.

**Objective:**

To explore the attitudes surrounding cancer pain among patients, carers, and health professionals at a tertiary cancer hospital.

**Methods:**

A cross‐sectional online survey was conducted at the Peter MacCallum Cancer Centre. The survey included demographic measures and statements assessing attitudes toward cancer pain management. Data was analyzed using descriptive statistics in IBM SPSS 29. Ethics approval was granted by the Peter MacCallum HREC.

**Results:**

308 participants (153 patients and carers, 155 health professionals) completed the survey. The results revealed significant variability in attitudes surrounding cancer pain and its management. Discrepancies in understanding between health professionals and patients/carers were observed. Differing views on the goals of pain management were revealed, with 51.6% of patients/carers expecting pain elimination compared to 20.6% of health professionals. The roles of clinicians in pain management were also perceived differently, highlighting a lack of clarity in responsibilities. Both groups emphasized the need for increased education on cancer pain and its management.

**Conclusions:**

The study revealed substantial variability in attitudes toward cancer pain management among patients, carers, and health professionals. Discrepancies emerged in understanding, with many patients and carers uncertain about the nature of cancer pain, contrasting with health professionals' recognition of its complexity. The terminology distinguishing “cancer pain” from “non‐cancer pain” may contribute to this confusion, suggesting a need to reconsider these semantics. Divergent views on clinician roles and opioid use underscored uncertainties, especially regarding specialist access and prescribing practices. Both groups emphasized the need for education to bridge these gaps, with clearer communication and revised guidelines potentially improving patient outcomes.

## INTRODUCTION

Cancer pain is defined by the International Association for the Study of Pain (IASP) as any pain arising from the primary tumor, its metastases, or associated treatments and is a prevalent, debilitating symptom. This phenomenon affects over 50% of all cancer patients^1^ and significantly impacts patients' quality of life.^2^, ^3^, ^4^ Despite its prevalence and impact, the distinction between cancer pain and non‐cancer pain is obscure.^5^ Historically, this distinction arose as a byproduct of the World Health Organization (WHO)'s analgesic step ladder, which aims to provide adequate pain relief to cancer patients through opioid provision.^6^ However, more recently the field of pain medicine has shifted toward managing cancer pain by addressing all biopsychosocial factors that contribute to a patient's pain experience.^7^, ^8^ These diverse and often conflicting factors contribute to the complexity surrounding cancer pain management.

Despite various existing approaches, effective pain management remains challenging in cancer care due to several barriers. These include the stigma associated with opioid use, a key component of pharmacological management for moderate‐to‐severe cancer pain.^9^, ^10^ This stigma is further amplified by findings in primary care, where clinicians are reluctant to prescribe opioids due to discomfort and fear of difficult conversations surrounding problematic opioid use disorder.^11^ Additionally, the ambiguous roles of different clinicians in managing cancer pain pose further obstacles.^12^, ^13^, ^14^, ^15^, ^16^, ^17^ This lack of clarity around specialist roles blurs the lines of responsibility in terms of opioid stewardship.

Another barrier to cancer pain management is the diverse attitudes toward pain management among healthcare professionals, patients, and carers.^18^, ^19^, ^20^, ^21^, ^22^, ^23^, ^24^, ^25^, ^26^, ^27^ Existing research has often focused on surrogate measurement of attitudes by knowledge of cancer pain and its management.^18^, ^19^, ^20^, ^21^, ^26^, ^27^ A limitation of these studies is that they lack the nuance required to explore such a complex experience, encompassing not only knowledge but also perceptions, biases, and past experiences. This angle was explored in a recent qualitative study,^28^ which analyzed the experience of patients with cancer pain. Challenges emerged surrounding explaining cancer pain, and strategies and barriers to management, as well as responsibilities of clinicians in cancer pain management were uncovered. It is hypothesized that there will be significant variability in attitudes surrounding cancer pain and its management, and this study has the potential to advance our understanding of the complex dynamics surrounding cancer pain management.

This study aimed to address this gap in the literature by exploring the attitudes of patients, carers, and healthcare professionals toward pain and its management at a tertiary cancer hospital, which has not been commonly explored in the literature. By conducting a cross‐sectional survey, data were gathered on these attitudes, including perceptions of pain, beliefs about pain management options, and attitudes toward healthcare professionals involved in pain care. A more detailed understanding of attitudes will inform areas for further exploration, help clinicians' understanding of cancer pain semantics, and contribute to improved cancer pain management outcomes.

## METHODS

### Study Design & Setting

A cross‐sectional exploratory survey design was adopted to allow for analysis of the frequency of responses. This was a single‐site study and recruited health professionals, patients, and carers at Peter MacCallum's Parkville campus. The Peter MacCallum Cancer Centre is a tertiary comprehensive cancer specialist hospital, located in Melbourne, Victoria, Australia. Data were collected from 22 March 2024 to 27 June 2024. This study was granted ethical approval by Peter MacCallum's HREC Committee (HREC/103563/PMCC).

The online survey was designed drawing from clinically derived questions in the pain service at Peter MacCallum Cancer Centre, as well as an extensive review of the literature concerning attitudes toward cancer pain management. The original survey format was inspired by an Australian survey of palliative care clinicians that looked at specialist pain service involvement in cancer pain management.^23^ Initial items were categorized into 2 surveys: one for health professionals and one for patients and carers. Language was altered for appropriateness, and the surveys were piloted among 10 individuals representative of the target population. Adaptations were made according to feedback.

The survey was hosted on Peter MacCallum Cancer Centre's RedCap database and consisted of two parts. The first section included demographic measures including age, gender, indigenous status, and area of profession (in health professionals only, see Appendix S2). The second section included measures regarding specific attitudes toward cancer pain management (see Appendices S3 and S4). The survey was completed on participants' personal devices, such as phones or computers or, in the case of Peter Mac staff, hospital computers. The survey took approximately 5 minutes to complete.

### Participants

The survey was open to health professionals, patients, and carers at Peter MacCallum Cancer Centre's Parkville Campus. Participants were taken to tailored surveys depending on whether they were health professionals or patients/carers.

Potential participants were excluded if they were aged under 18, were non‐English speaking, and/or did not have access to a personal device capable of scanning a QR code, due to the self‐directed online nature of accessing the survey.

### Measures

Initial demographic questions were followed by Likert scale questions. Respondents were asked to select a measure of agreement along a three‐point scale of agree, unsure, and disagree. Specifically, statements explored areas such as understanding of cancer pain, perceived role of clinicians, opinions regarding the use of opioid medications, and need for education.

In total, there were four demographic questions for health professionals and three in the visitor survey. There were 20 questions measuring attitudes in the visitor survey and 22 questions in the health professional survey (see Appendices S3 and S4, respectively).

### Procedure

A voluntary sampling approach was employed. Health professionals were recruited by invitation in person and via email. Visitors to Peter MacCallum Cancer Centre were recruited via posters placed in waiting areas around Specialist Clinics and Outpatient Pathology.

A preamble contained an introduction to the study's aim and significance, as well as instructions for completing the following section (see Appendix S1). Implied consent was obtained by progressing through the survey. No identifiable data was collected, and confidentiality and anonymity were maintained throughout. Participants were asked to respond with their perspective (agree, unsure, disagree) on provided statements. Responses to statements were recorded and saved in real time to the database. The RedCap database used for data collection and subsequent analysis featured safeguarding measures built in, and access was limited to the investigators through a secure password.

### Sample size

Sample size was based on feasibility. Due to limitations in time as well as adoption of a voluntary sampling approach, it was deemed that a sample size of 150 health professionals and 150 visitors (for a total sample size of 300) was feasible.

### Statistical analysis

Data were extracted from Peter Mac's RedCAP database into SPSS 29 (IBM) for analysis. Descriptive statistical analysis of categorical data was undertaken, including the number of responses and percentages of frequency to generate insight into attitudes toward cancer pain management.

Cohort characteristics were gathered by items relating to factors such as age, gender, indigenous status, and area of profession (for health professionals only). The surveys used in the study do not produce a total score; therefore, analysis focused on the frequencies of individual questions. The number of respondents that commenced the survey but did not complete it was reported. Incomplete survey responses were excluded from statistical analysis.

## RESULTS

**FIGURE 1:**
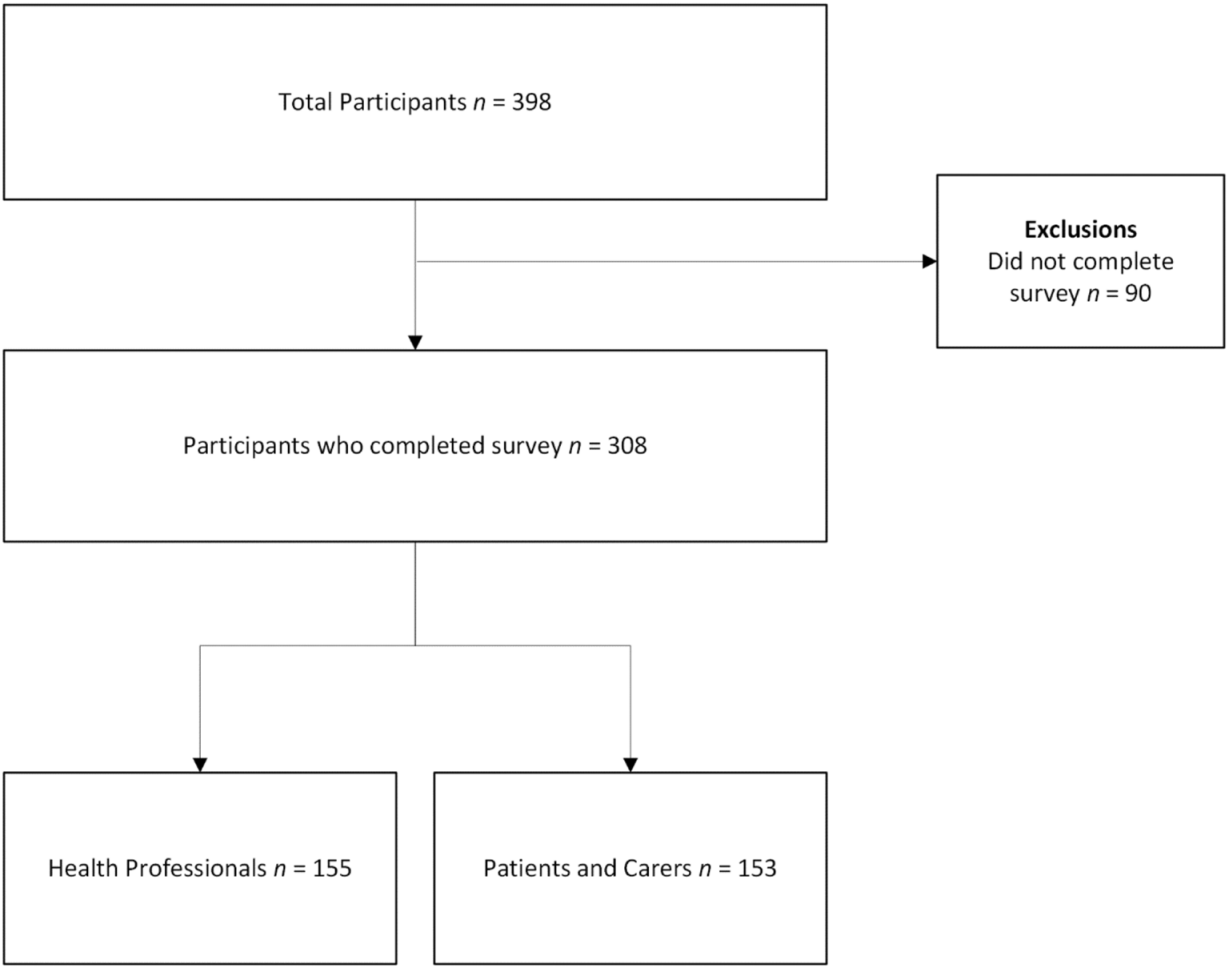
Participant flow diagram illustrating recruitment and exclusions from the analysis.

From 398 participants who accessed the survey, a total of 308 participants completed the survey, including 153 patients and carers and 155 health professionals (see Figure 1). Data from incomplete surveys was excluded from analysis. Participants' ages ranged from 18 to over 70, with the largest proportion falling between 50 and 70 in the patient and carer group, compared with a majority in the health professional group falling between 30 and 50. Sample characteristics are presented in Tables 1 and 2.

**TABLE 1:** Sample characteristics for Patients and Carers (*N* = 153).

	*N* (%)
Sex	
Male	71 (46.4)
Female	82 (53.6)
Non‐binary	1 (0.6)
Prefer not to say	1 (0.6)
Age	
18–30	12 (7.8)
31–50	44 (28.8)
51–70	62 (40.5)
>70	35 (22.9)
Identifies as first nations person	
Yes	4 (2.6)
No	146 (95.4)
Prefer not to say	3 (2.0)

**TABLE 2:** Sample characteristics for Health Professionals (*N* = 155).

	*N* (%)
Sex	
Male	53 (34.2)
Female	100 (64.5)
Non‐binary	1 (0.6)
Prefer not to say	1 (0.6)
Age	
18–30	37 (23.9)
31–50	82 (52.9)
51–70	32 (20.6)
>70	4 (2.6)
Identifies as first nations person	
Yes	3 (1.9)
No	151 (97.4)
Prefer not to say	1 (0.6)
Area of Profession	
Nurse	48 (31.0)
Administration	27 (17.4)
Anesthetist	15 (9.7)
Radiation Oncologist	15 (9.7)
Medical Oncologist	14 (9.0)
Allied Health Clinician	8 (5.2)
Surgical Oncologist	7 (4.5)
Pharmacist	5 (3.2)
Palliative Care Clinician	3 (1.9)
Internal Medicine Clinician	2 (1.3)
Medical Student	2 (1.3)
HMO	2 (1.3)
Doctor	2 (1.3)
Dentist	2 (1.3)
Pain Specialist	2 (1.3)
GP	1 (0.6)

A majority of participants were female in both groups (64.5% of health professionals and 53.6% of patients and carers). In both samples, there was a proportion of First Nations persons (1.9% and 2.6% in the health professional and patient and carer groups, respectively); 1.3% of participants elected not to disclose their First Nations status. The largest group of health professionals that responded to the survey was nurses (31.0%), followed by administration (17.4%).

### Responses from patients and Carers

Responses to the survey among the sample of patients and carers are summarized in Table 3.

**TABLE 3:** Response numbers and frequencies of items in the survey for patients and carers.

	Item	Agree	Unsure	Disagree
		*N* (%)	*N* (%)	*N* (%)
1	Cancer pain feels different from other types of pain.	56 (36.6)	75 (49.0)	22 (14.4)
2	Pain that arises from cancer treatment is considered cancer pain.	80 (52.3)	51 (33.3)	22 (14.4)
3	Pain only manifests from the tumor itself.	20 (13.1)	60 (39.2)	73 (47.7)
4	Pain specialists only manage non‐cancer pain.	13 (8.5)	63 (41.2)	77 (50.3)
5	Managing cancer pain is complex.	109 (71.2)	38 (24.8)	6 (3.9)
6	Oncology teams are effective at managing cancer pain.	94 (61.4)	45 (29.4)	14 (9.2)
7	Exercise, counseling and other non‐medication‐based methods can be used to manage cancer pain.	109 (71.2)	33 (21.6)	11 (7.2)
8	Accessing tools to manage cancer pain (like medicine, counseling, and exercise) is an easy process for patients.	65 (42.5)	53 (34.6)	35 (22.9)
9	Cancer pain can be treated with various approaches.	131 (85.6)	21 (13.7)	1 (0.7)
10	Allied health should be included in the management of cancer pain (i.e., physio, psych support, occupational therapy, etc).	130 (85.0)	21 (13.7)	2 (1.3)
11	Cancer is always associated with pain.	28 (18.3)	45 (29.4)	80 (52.3)
12	The goal of pain management is to eliminate pain.	79 (51.6)	26 (17.0)	48 (31.4)
13	Explaining cancer pain is challenging.	101 (66.0)	40 (26.1)	12 (7.8)
14	Patients and carers need education around cancer pain management.	139 (90.8)	13 (8.5)	1 (0.7)
15	Medications used to manage cancer pain are regulated. Regulations for pain relief (like opioids) should be less strict for cancer patients compared to others.	86 (56.2)	42 (27.5)	25 (16.3)
16	Any doctor should be able to prescribe strong opioid medications for cancer pain, when necessary.	87 (56.9)	38 (24.8)	28 (18.3)
17	All cancer patients experiencing pain should be engaged with a Palliative Care service for pain management.	54 (35.3)	58 (37.9)	41 (26.8)
18	Cancer pain management should be managed by the Palliative Care department, only.	7 (4.6)	54 (35.3)	92 (60.1)
19	There is a need for education about cancer pain within the community.	122 (79.7)	23 (15.0)	8 (5.2)
20	GPs are hesitant in prescribing opioid pain relief.	89 (58.2)	62 (40.5)	2 (1.3)

For patients and carers, there was notable variation in understanding cancer pain. While 71.2% agreed that managing cancer pain is complex, nearly half (49%) were unsure whether cancer pain feels different from other types of pain, indicating a lack of clarity in their understanding. Furthermore, 66% agreed that explaining their pain was challenging, underscoring the communication difficulties in conveying their experience. Regarding the role of clinicians, 61.4% agreed that oncology teams were effective in managing pain, though a substantial portion (34.6%) was unsure about how to access appropriate pain management tools. When it came to the involvement of palliative care teams, 60.1% disagreed with the idea that pain management should be solely under their purview, yet 37.9% remained unsure about the extent of palliative care's involvement. On the topic of opioid use, 56.9% agreed that any doctor should be able to prescribe strong opioids for cancer pain, but 58.2% perceived general practitioners (GPs) as hesitant to do so. Additionally, opinions were divided on the goal of pain management, with 51.6% of patients and carers agreeing that pain elimination should be the objective, while 31.4% disagreed. Finally, there was near‐unanimous support for the need for education, with 90.8% agreeing that patients and carers require more education about cancer pain management, and 79.7% also agreeing that the community at large needs further education on this issue.

### Responses from health professionals

Responses to the survey among the sample of patients and carers are summarized in Table 4.

**TABLE 4:** Response numbers and frequency percentages to items in the survey for health professionals.

	Item	Agree	Unsure	Disagree
		*N* (%)	*N* (%)	*N* (%)
1	Cancer pain is unique and different from other forms of non‐cancer pain.	124 (80.0)	21 (13.5)	10 (6.5)
2	Pain resulting from cancer treatment is also considered cancer pain.	113 (72.9)	23 (14.8)	19 (12.3)
3	The only source of cancer pain is from the tumor itself.	7 (4.5)	11 (7.1)	137 (88.4)
4	Managing cancer pain is complex.	150 (96.8)	3 (1.9)	2 (1.3)
5	Non‐pharmacological (physio, OT, mindfulness, etc.) pain management modalities can also alleviate cancer pain.	140 (90.3)	13 (8.4)	2 (1.3)
6	A step ladder approach should be the basis of opioid analgesic escalation in management of cancer pain.	111 (71.6)	32 (20.6)	12 (7.7)
7	The use of solely pharmacological analgesia is enough to achieve managed cancer pain.	8 (5.2)	22 (14.2)	125 (80.6)
8	A multidisciplinary (medical and allied health) approach to cancer pain management is gold standard care.	145 (93.5)	8 (5.2)	2 (1.3)
9	Pain specialists only manage non‐cancer pain.	3 (1.9)	13 (8.4)	139 (89.7)
10	It is difficult to refer to a pain specialist.	31 (20.0)	50 (32.3)	74 (47.7)
11	Cancer is always associated with pain.	21 (13.5)	14 (9.0)	120 (77.4)
12	The goal of pain management is to eliminate pain.	32 (20.6)	8 (5.2)	115 (74.2)
13	Cancer pain can be properly assessed formulaically.	23 (14.8)	64 (41.3)	68 (43.9)
14	Patients and carers need pain education to understand and manage cancer pain effectively.	143 (92.3)	9 (5.8)	3 (1.9)
15	Cancer pain patients should have to abide by the same regulations in regards to accessing opioids as non‐cancer pain patients.	40 (25.8)	48 (31.0)	67 (43.2)
16	Under the current regulatory systems (safe script, limited supplies, etc.), accessing pain relief is fair for non‐palliative cancer patients.	45 (29.0)	84 (54.2)	26 (16.8)
17	Strong opioid medications should be easily accessible for people experiencing cancer pain.	102 (65.8)	31 (20.0)	22 (14.2)
18	Access to a pain specialist is a barrier to cancer pain management.	67 (43.2)	53 (34.2)	35 (22.6)
19	Palliative Care teams play a central role in the comprehensive management of cancer pain.	135 (87.1)	15 (9.7)	6 (3.2)
20	All cancer patients experiencing pain should be engaged with a Palliative Care service for pain management.	49 (31.6)	40 (25.8)	66 (42.6)
21	There is a need for general education surrounding opioid medication in the community.	140 (90.3)	13 (8.4)	2 (1.3)
22	Primary health professionals are hesitant in prescribing opioid analgesia.	100 (64.5)	44 (28.4)	11 (7.1)

Among health professionals, in terms of understanding of cancer pain, a large majority (96.8%) agreed that managing cancer pain is complex, and 80% agreed that cancer pain differs from other types of pain. However, there was some divergence in views on the nature of pain, with only 4.5% agreeing that the sole source of cancer pain is the tumor itself, and the rest either disagreeing (88.4%) or being unsure (7.1%). Regarding the roles of clinicians, 89.7% of health professionals agreed that pain specialists should manage cancer pain, and 87.1% acknowledged that palliative care teams play a central role. However, opinions were split on whether palliative care should exclusively manage cancer pain, with 42.6% disagreeing and 31.6% agreeing. Access to pain specialists was viewed as a potential barrier, with 43.2% agreeing that it is difficult to refer patients to these specialists. When it came to opioid use, 64.5% of health professionals agreed that GPs are hesitant to prescribe opioids, reflecting concerns about the complexities of opioid prescription. There was also uncertainty about whether current opioid regulations are fair for cancer patients, with 54.2% being unsure and 29% agreeing that access is fair. Notably, only 20.6% of health professionals agreed with the goal of complete pain elimination, with the majority (74.2%) disagreeing, indicating a divergence from patient expectations. Lastly, there was strong consensus on the need for education, with 92.3% agreeing that patients and carers require more education about cancer pain management, and 90.3% advocating for increased community education on opioid use to better inform the public and reduce stigma.

## DISCUSSION

This study investigated the attitudes toward pain management among patients and carers, as well as health professionals, at a cancer hospital. The survey of 308 patients, carers, and health professionals at Peter MacCallum exemplified that there exists substantial variation in views and attitudes toward pain management in cancer care and is potentially reflective of overarching societal views. This study contributes valuable insights into the attitudes and beliefs surrounding pain management in cancer care at Peter MacCallum Cancer Centre. The demographic distribution of participants generally reflected that of the Australian population in terms of age,^29^ gender^30^ and indigenous status^31^ and these findings can hence be extrapolated to the broader community.

### Ambiguity in attitudes toward cancer pain management

Overall, significant ambiguity surrounding attitudes toward cancer pain management was observed. There was a considerable discrepancy in the understanding of cancer pain between the two groups surveyed. Health professionals generally recognized cancer pain as a distinct subset of pain (80.0% agreed) and considered treatment‐associated pain as part of this subset (72.9% agreed). Interestingly, however, a substantial proportion (49.0% and 33.0%, respectively) of patients and carers remain unsure about these aspects. It is worth considering whether this distinction of terms is beneficial for patient outcomes. This ambiguity among the patient and carer group may stem from the semantic division of pain into ‘cancer pain’ and ‘non‐cancer pain’. The results of this study seem to lend support to the argument posited by Peppin and Schatman that this distinction of terms fosters challenges in pain management, due to its philosophical, rather than biomedical basis.^5^ They posit that language possesses the ability to stigmatize and marginalize patients.

Interestingly, there was incongruity in beliefs surrounding the goals of pain management between health professionals and patients and carers. The difference in opinion was notable, with 51.6% of patients of the belief that pain management should aim to eliminate pain completely, contrasting with the 20.6% of health professionals who agreed with the same statement. This divide in understanding may lead to, or stem from, mismatched expectations between clinicians and patients.^32^ Previous research has identified mismatches in expectation between patients and clinicians^33^, ^34^, ^35^, ^36^ which may arise from a clash of patients' desire for a more personal healthcare journey with the healthcare system‐oriented goals of clinicians.^37^ Further complexity may be attributed to emphasis on pain as the “5th vital sign,” an initiative that gained momentum in the early 2000s. While it promoted routine pain assessment to improve patient outcomes, it may have unintentionally reinforced the belief that complete pain relief is always achievable.^38^, ^39^ Studies have shown that this focus on pain as a vital sign has created a challenge for clinicians, who are more likely to prioritize realistic, individualized pain control over total elimination.^40^ As a result, patients often enter clinical encounters with expectations shaped by these initiatives, leading to potential dissatisfaction when their pain is not fully relieved.

Health professionals were notably divided regarding the use of formulae for pain assessment, without consideration of the multitude of biopsychosocial factors that make up patients' pain experiences. 41.3% of respondents were unsure regarding their opinion on this statement, and 43.9% disagreed. This may reflect different preferences in pain management strategies, which are a product of the plethora of approaches available^41^ or also a biomedical‐only approach to pain rather than a complex formula‐based approach. The variability among responses between and within groups highlights the discordance in attitudes surrounding cancer pain and is reflective of the heterogeneous and multifaceted nature of cancer pain.

### Roles and responsibilities of clinicians

The contrasting perceptions of clinician roles in cancer pain management emphasize a lack of clarity surrounding responsibilities in this area. Significant discordance in the perception of roles and responsibilities of clinicians was revealed. While a proportion of patients and carers (42.5%) reported ease in accessing pain management tools and clinicians, a notable percentage (34.6%) remained uncertain, indicating a potential gap in understanding. Notably, a majority (47.7%) of health professionals disagreed that referring patients to a pain specialist is difficult, yet a significant proportion (43.2%) acknowledged limited access to pain specialists as a barrier to effective pain management. This suggests a potential underestimation of the challenges faced in navigating pain management in cancer care or may also reflect institutional factors and past experiences when referring someone with pain.

Moreover, health professionals overwhelmingly recognized that pain specialists are involved in managing cancer pain (89.7% agreed), juxtaposing the uncertainty observed among the sample of patients and carers, with only 60% of patients and carers agreeing with the statement “oncology teams are effective at managing cancer pain.” This is consistent with the findings of previous research,^42^ and may suggest that further teaching of pain medicine is needed in Australian medical schools, a notion supported in the literature.^43^ Further contributing complexity to this issue is the increasing numbers of cancer survivors,^44^ which results in patients entering a position of ambiguity in their cancer journey and further blurring the lines of responsibility among healthcare providers.

Additionally, the observed uncertainty regarding the role of specialists in cancer pain management highlights the need for clearer roles and responsibilities of pain specialists and other clinicians involved in cancer care. In responses from both groups, no clear stance was observed as to whether all cancer patients should be engaged with a palliative care service. This echoes previous research that has explored the uncertainty surrounding clinician responsibilities regarding pain management.^28^, ^35^ Overall, these findings highlight the lack of defined roles and responsibilities in pain management, which is a product of the complexity associated with managing cancer pain. Further nuanced exploration is required to identify and improve the distinction of these roles to improve the management of patients' cancer pain.

### Attitudes toward opioids in cancer pain management

The past decade has seen great increases in media attention surrounding opioid use, and regulations surrounding opioid prescription for pain management have seen changes in legislation, especially in Australia.^45^ Over time, opioid prescription has been reliant on broad regulations which have swung from overly liberal to more restricted; however, nuance and a balanced approach are necessary, a notion that is supported in the literature.^46^, ^47^

This study explored the topic of opioid use and prescription attitudes in the context of cancer pain management. The reluctance of General Practitioners to prescribe opioid medication, as perceived by 64.5% of health professionals and 58.2% of patients/carers, presents a barrier to effectively managed pain and is reflective of previous research findings.^48^ Other studies have revealed that hesitation may stem from concerns about addiction or a greater need for expertise regarding pain management guidelines.^11^ It is worth noting, however, that there exists variation in prescribing behaviors among clinicians. Previous research has indicated that clinicians cite factors such as “overthinking” perception by their peers as influencing their opioid prescribing behaviors in cancer pain, as well as fearing the consequences of their prescribing decisions.^35^ It is this complexity that must be considered in the practical implementation of prescribing guidelines.

Interestingly, there was a mixed sentiment regarding the regulation of opioid medication for cancer patients. 56.2% of health professionals were unsure with the fairness of current regulations, comparable with 54.2% of patients and carers who were unsure. This may be reflective of the polarization in pain management, both in the field of pain medicine as well as in the public eye.^49^, ^50^ Responses to the item, “Any doctor should be able to prescribe opioids,” reflect a sentiment toward increased ease of access to these medications in the context of cancer pain. This sentiment may stem from concerns about potential barriers to adequate pain relief,^51^ however, it is crucial to balance these competing needs for access with the needs for responsible opioid stewardship practices to prevent misuse, addiction, and other adverse outcomes,^52^, ^53^ while simultaneously improving pain outcomes.

### Need for education surrounding cancer pain

Moreover, a strong consensus emerged among both samples regarding the need for education on pain management; 90% of both groups emphasized the importance of educational initiatives for patients and carers as well as primary care clinicians. This overwhelming agreement supports previous findings that identified links in pain education with decreased post‐operative acute pain.^54^ Further exploration is, however, required to identify if this is also the case in cancer pain. By educating patients and carers around the multifaceted nature of cancer pain management, clinicians can help bridge this gap in understanding and foster more realistic expectations for pain control,^55^ as well as decrease fear, which has been associated with improved outcomes.^56^ This, in conjunction with improved communication, may lead to increased patient satisfaction and better pain management outcomes, as highlighted in previous research.^57^, ^58^

### Limitations

Several limitations warrant consideration. Analysis of the “Unsure” responses, as a proportion of total responses, could have revealed whether the questions were unclear or if there is a greater need for education on these topics. Additionally, translating the survey into other languages may have increased inclusivity and revealed cultural nuances in pain management perceptions. Offering a paper‐based survey may have also ensured inclusivity for participants without access to QR‐capable devices. Collection of demographic data that distinguished between patients and carers may have provided further insights into their unique perspectives and experiences with cancer pain and its management. An additional limitation of this study is that the survey items are not identical for the patient/carer and health professional surveys, limiting the extent to which comparisons can be made in terms of response frequencies.

### Future directions

These findings highlight areas for more in‐depth analysis, such as expectations in pain management of patients and caregivers compared with clinicians. Additionally, mixed sentiments regarding opioid regulation warrant further exploration to understand the underlying concerns and beliefs that shape these attitudes. There was a shared recognition of the complexity of managing cancer pain, which may be a valuable commonality for adapting and matching expectations between patients/carers and clinicians. By gaining a deeper understanding of these factors, researchers and policymakers can develop evidence‐based guidelines and educational resources that address concerns, reduce stigma, and promote safe and effective opioid use for cancer pain management.

Moreover, the study's findings regarding barriers to accessing pain specialists and the perceived reluctance of primary care clinicians to prescribe opioids underscore the need for targeted interventions to improve access and address gaps in knowledge. Additionally, these findings bring to light discussions surrounding a paradigm shift in pain medicine. For pain management outcomes, it may be more favorable to move from semantic distinctions between “cancer pain” and “non‐cancer pain” toward distinguishing pain in terms of whether it is acute, chronic, or end‐of‐life. Future research could focus on developing educational programs for clinicians on pain management guidelines and appropriate opioid prescription. Additionally, investigating differences in the attitudes of patients and carers may uncover further areas for exploration. By addressing these specific key findings and exploring potential solutions, future research can contribute to the improvement of pain management approaches and, ultimately, better patient outcomes.

### Conclusion

This study identified significant variability in attitudes toward cancer pain management among patients, carers, and health professionals. Key findings revealed discrepancies in the understanding of cancer pain, with health professionals generally recognizing it as a distinct and complex experience, while a significant portion of patients and carers were uncertain about its nature. The semantics of pain, specifically the distinction between “cancer pain” and “non‐cancer pain,” may have contributed to this confusion. Such terminology, while intended to categorize pain types, can obscure patient understanding and lead to challenges in management, potentially fostering unrealistic expectations regarding pain outcomes.

In terms of the perceived role of clinicians, health professionals favored a multidisciplinary approach, though patients and carers showed uncertainty about accessing the necessary tools for pain management and the roles of various specialists. Opinions regarding the use of opioid medications highlighted a shared concern about GP reluctance to prescribe opioids, with both groups acknowledging the complexities of opioid use and regulation in cancer care. Finally, there was a unanimous recognition of the need for education to bridge gaps in understanding and to align the expectations of both patients and clinicians. This, coupled with clearer communication about the nature of cancer pain, could help reduce stigma and improve pain management strategies, ultimately leading to better patient outcomes.

## AUTHOR CONTRIBUTIONS

The authors confirm contribution to the paper as follows: study conception: Young, J.; study design: Henriksen, E., Young, J., Power, C., Chan, C.; data collection: Henriksen, E.; analysis and interpretation of results: Henriksen, E., Young, J., Power, C., Chan, C.; draft manuscript preparation: Henriksen, E. All authors reviewed the results and approved the final version of the manuscript.

## CONFLICT OF INTEREST STATEMENT

All authors declare that they have no conflicts of interest.

## PATIENT CONSENT STATEMENT

This study excluded participants below the age of 18; therefore, parental/guardian consent was not required to be obtained.

## Supporting information


Appendix S1.

Appendix S2.

Appendix S3.

Appendix S4.

## Data Availability

The data that support the findings of this study are available from the corresponding author upon reasonable request.

## References

[1] Snijders S , van den Beuken‐ Everdingen MHJ , de Rijke JM , Schouten HC , van den Bemt PMLA . Prevalence of pain in patients with cancer: a systematic review and meta‐analysis. J Natl Compr Canc Netw. 2023;21(7):787–800.37549909

[2] Greco C , Roberto A , Corli O , Deandrea S , Bandieri E , Cavuto S . Quality of cancer pain management: an update of a systematic review of undertreatment of patients with cancer. J Clin Oncol. 2014;32(36):4149–4156. 25403222 10.1200/JCO.2014.56.0383

[3] Sanford SD , Moore TW , Fanciullo GJ , Wehby GL . The prevalence of chronic pain in cancer survivors: a systematic review and meta‐analysis. Pain Med. 2019;20(11):2209–2222.

[4] Jiang H , Chen Y , Zhang Y , Hu L , Hu J , Zhang B . Chronic pain in cancer survivors: a cross‐sectional survey in a Chinese population. J Pain Symptom Manage. 2019;58(5):900–907.

[5] Peppin JF , Schatman ME . Terminology of chronic pain: the need to “level the playing field”. J Pain Res. 2016;9:23–24. 26869809 10.2147/JPR.S99629 PMC4734783

[6] Ventafridda V , Saita L , Ripamonti CI , De Conno F . WHO guidelines for the use of analgesics in cancer pain. Int J Tissue React. 1985;7(1):93–96. 2409039

[7] Gatchel R , Peng Y , Peters M , Fuchs P , Turk D . The biopsychosocial approach to chronic pain: scientific advances and future directions. Psychol Bull. 2007;133:581–624. 17592957 10.1037/0033-2909.133.4.581

[8] Bäckryd E . Should cancer pain still be considered a separate category alongside acute pain and chronic non‐cancer pain? Reflections on ICD‐11. Front Pain Res. 2024;5:1397413. 10.3389/fpain.2024.1397413 PMC11096455 38756912

[9] Harsanyi S , Richert T , Sittl R . Opioid stigma: a narrative review of concepts, assessment tools and interventions. J Pain Res. 2023;16:2867–2881.

[10] Webster F , Rice K , Sud A . A critical content analysis of media reporting on opioids: the social construction of an epidemic. Soc Sci Med. 2020;244:112642. 31731136 10.1016/j.socscimed.2019.112642

[11] Wilson H , Roxas BH , Lintzeris N , Harris M . Diagnosing and managing prescription opioid use disorder in patients prescribed opioids for chronic pain in Australian general practice settings: a qualitative study using the theory of planned behaviour. BMC Prim Care. 2024;25(1):236. 38961328 10.1186/s12875-024-02474-6 PMC11223276

[12] Page K . Integration of pain and palliative care services in India: results from a cross‐sectional survey among palliative care physicians. Indian J Palliat Care. 2017;23(3):231–237.28827924

[13] Pai M , Yadav R , Kumar S . Current scenario of pain medicine practice in India: results from a national survey. Indian J Pain. 2021;35(2):118–125.

[14] Linklater J , Davies A , Brull R . Pain and palliative care services in the UK 2002: a national survey. Palliat Med. 2002;16(4):289–298. 10.1191/0269216302pm535oa 12380662

[15] Kay J , Davies A , Brull R . Pain and palliative care services in the UK 2007: a national survey. Palliat Med. 2007;21(8):695–703.

[16] Acheon Working Group . Prevalence of cancer pain in Asian adult cancer patients: a cross‐sectional study from the ACHEON pain and palliative care working group. Pain Res Manag. 2015;20(2):81–87.

[17] Breuer B , Fleishman S , Portenoy RK . Pain management by oncologists: a national survey of medical oncologists in the united StatesBreuer. J Clin Oncol. 2015;33(8):881–887.

[18] Wazqar D . Evaluating Saudi nursing Students' knowledge and attitudes toward cancer pain management: implications for nursing education. Saudi Med J. 2019;26(2):61–70.

[19] Zaabi S , Al‐Balushi A , Al‐Magbali M , Al‐Sinani S , Al‐Zakwani I . Assessing Nurses' knowledge and attitudes towards cancer pain Management in Oman. Cancers Basel. 2023;15(15):3925. 37568741 10.3390/cancers15153925 PMC10417855

[20] Bouya S , Ouassou H , Bernoussi A . Knowledge and attitudes of nurses toward cancer pain management: a systematic review. Pain Manag Nurs. 2019;20(1):71–79.

[21] Kasasbeh A , Al‐Hussami M , Alzoubi F . Knowledge and attitudes of health professionals toward cancer pain management: a systematic review. Pain Manag Nurs. 2017;18(4):252–260.

[22] Luckett T , Phillips J , Lovell M . Pain management in cancer: perspectives of Australian oncologists. Med J Aust. 2014;200(10):582–586.24882489

[23] Lovell M , Agar M , Luckett T , Davidson PM , Green A , Clayton J . Australian survey of current practice and guideline use in adult cancer pain assessment and management: perspectives of palliative care physicians. J Palliat Med. 2013;16(11):1403–1409. 24168350 10.1089/jpm.2013.0245 PMC3822364

[24] Phillips J , Lovell M , Luckett T , Agar M , Green A , Davidson P . Australian survey of current practice and guideline use in adult cancer pain assessment and management: the community nurse perspective. Collegian. 2015;22(1):33–41. 26285407 10.1016/j.colegn.2013.11.002

[25] Bender CM , Oberle K , Schumacher K . What do patients with cancer want to know about pain? Oncol Nurs Forum. 2008;35(4):669–675.

[26] Lou XM , Shang SS . Attitudes towards pain management in hospitalized cancer patients and their influencing factors. Chin J Cancer Res. 2017;29(1):75–85. 28373756 10.21147/j.issn.1000-9604.2017.01.09 PMC5348478

[27] Makhlouf K , Davis MP , McMillan SC . Attitudes towards cancer pain among patients, caregivers, and healthcare professionals: a systematic review and meta‐analysis. J Clin Oncol. 2020;38(15):1670–1681.

[28] Ikander T , Raunkiær M , Voetmann C , Pedersen CV , Jarlbaek L . Cancer‐related pain experienced in daily life is difficult to communicate and to manage–for patients and for professionals. Scand J Pain. 2024;24(1):20230107. 10.1515/sjpain-2023-0107 38776518

[29] Health AIo , Welfare . Profile of Australia's population. Canberra: AIHW; 2024.

[30] Australian Bureau of Statistics . Population: Census. Canberra, ACT: ABS; 2021.

[31] Australian Institute of Health and Welfare . Profile of first nations people. Canberra: AIHW; 2024.

[32] Calpin P , Imran A , Harmon D . A comparison of expectations of physicians and patients with chronic pain for pain clinic visits. Pain Pract. 2017;17(3):305–311. 26992011 10.1111/papr.12428

[33] Friele R , Reitsma P , De Jong J . Complaint handling in healthcare: expectation gaps between physicians and the public; results of a survey study. BMC Res Notes. 2015;8:1–7. 26429097 10.1186/s13104-015-1479-z PMC4591727

[34] Bouwman R , Bomhoff M , Robben P , Friele R . Is there a mismatch between the perspectives of patients and regulators on healthcare quality? A survey study. J Patient Saf. 2021;17(7):473–482. 28857951 10.1097/PTS.0000000000000413

[35] Bulls HW , Hamm M , Wasilewski J , Olejniczak D , Bell SG , Liebschutz JM . “To prescribe or not to prescribe, that is the question”: perspectives on opioid prescribing for chronic, cancer‐related pain from clinicians who treat pain in survivorship. Cancer. 2024;130:3034–3042. 38567685 10.1002/cncr.35299

[36] Natafgi N , Ladeji O , Blackwell S , Hong YD , Graham G , Cort M , et al. Similar values, different expectations: how do patients and providers view ‘health'and perceive the healthcare experience? Health Expect. 2022;25(4):1517–1528. 35411659 10.1111/hex.13493 PMC9327836

[37] Oster A , Wiking E , Nilsson GH , Olsson CB . Patients' expectations of primary health care from both patients' and physicians' perspectives: a questionnaire study with a qualitative approach. BMC Prim Care. 2024;25(1):128. 38658808 10.1186/s12875-024-02389-2 PMC11040877

[38] Cohen MZ , Easley MK , Ellis C , Hughes B , Ownby K , Rashad BG , et al. Cancer pain management and the JCAHO's pain standards: an institutional challenge. J Pain Symptom Manage. 2003;25(6):519–527. 12782432 10.1016/s0885-3924(03)00068-x

[39] Levy N , Sturgess J , Mills P . Pain as the fifth vital sign; and dependence on the numerical pain scale is being abandoned in the US: why? Br J Anaesth. 2018;120(3):435–438. 29452798 10.1016/j.bja.2017.11.098

[40] Gordon DB , Rees SM , McCausland MP , Pellino TA , Sanford‐Ring S , Smith‐Helmenstine J , et al. Improving reassessment and documentation of pain management. Joint Comm J Qual Patient Saf. 2008;34(9):509–517. 10.1016/S1553-7250(08)34065-3 18792655

[41] Mestdagh F , Steyaert A , Lavand'homme P . Cancer pain management: a narrative review of current concepts, strategies, and techniques. Curr Oncol. 2023;30(7):6838–6858. 37504360 10.3390/curroncol30070500 PMC10378332

[42] Breuer B , Chang VT , Von Roenn JH , Gunten C , Neugut AI , Kaplan R , et al. How well do medical oncologists manage chronic cancer pain? A national survey. Oncologist. 2015;20(2):202–209. 25582140 10.1634/theoncologist.2014-0276 PMC4319627

[43] Shipton EE , Bate F , Garrick R , Steketee C , Visser EJ . Pain medicine content, teaching and assessment in medical school curricula in Australia and New Zealand. BMC Med Educ. 2018;18(1):110. 29751806 10.1186/s12909-018-1204-4 PMC5948674

[44] Health AIo , Welfare . Cancer data in Australia. Canberra: AIHW; 2023.

[45] Therapeutic Goods Administration . Prescription opioids: Information for health professionals: Therapeutic Goods Administration (TGA). 2022. Available from: https://www.tga.gov.au/resources/resource/guidance/prescription‐opioids‐information‐health‐professionals

[46] Rieder TN . There's never just one side to the story: why America must stop swinging the opioid pendulum. Narrative Inquiry Bioeth. 2018;8(3):225–231. 10.1353/nib.2018.0071 30595589

[47] Noreika D , Konecny M . The pendulum: the need to develop a safe, effective, and equitable management strategy for opioids in cancer patients. Risk Manag Healthc Policy. 2024;17:1079–1082. 10.2147/RMHP.S455252 38686131 PMC11057629

[48] Gill S , Bailey J , Nafees S , Poole R . A qualitative interview study of GPs' experiences of prescribing opioid medication for chronic pain. BJGP Open. 2022;6(4):BJGPO.2022.0085. 10.3399/BJGPO.2022.0085 36216370 PMC9904793

[49] Vargas‐Schaffer G , Cogan J . Attitudes toward opioids and risk of misuse/abuse in patients with chronic noncancer pain receiving long‐term opioid therapy. Pain Med. 2018;19(2):319–327. 28340165 10.1093/pm/pnw338

[50] Ebbert JO , Philpot LM , Clements CM , Lovely JK , Nicholson WT , Jenkins SM , et al. Attitudes, beliefs, practices, and concerns among clinicians prescribing opioids in a large academic institution. Pain Med. 2018;19(9):1790–1798. 29177439 10.1093/pm/pnx140

[51] Othman WM , Al‐Atiyyat N . Knowledge, perceived barriers, and practices of oncology nurses regarding cancer pain management. Electron J Gen Med. 2022;19(6)(1–7).

[52] Shrestha S , Khatiwada AP , Sapkota B , Sapkota S , Poudel P , Kc B , et al. What is “opioid stewardship”? An overview of current definitions and proposal for a universally acceptable definition. J Pain Res. 2023;16:383–394. 36798077 10.2147/JPR.S389358 PMC9926985

[53] Pattullo C , Suckling B , Donovan P , Hall L . Developing a framework for implementing opioid stewardship programmes in Australian hospital settings. Intern Med J. 2022;52(4):530–541. 34617378 10.1111/imj.15555

[54] Horn A , Kaneshiro K , Tsui BCH . Preemptive and preventive pain psychoeducation and its potential application as a multimodal perioperative pain control option: a systematic review. Anesth Analg. 2020;130(3):559–573. 31335400 10.1213/ANE.0000000000004319

[55] Rogers AH , Bakhshaie J , Orr MF , Ditre JW , Zvolensky MJ . Health literacy, opioid misuse, and pain experience among adults with chronic pain. Pain Med. 2020;21(4):670–676. 30938818 10.1093/pm/pnz062 PMC12104017

[56] Javdaneh N , Saeterbakken A , Shams A , Barati A . Pain neuroscience education combined with therapeutic exercises provides added benefit in the treatment of chronic neck pain. Int J Environ Res Public Health. 2021;18:8848. 34444594 10.3390/ijerph18168848 PMC8394804

[57] Ma X , Chen R , Li W , Huang P . A systematic review and meta‐analysis of pain neuroscience education for chronic low back pain: short‐term outcomes of pain and disability. Physiother Theory Pract. 2023;40:2130–2149. 37395152 10.1080/09593985.2023.2232003

[58] Costa N , Blyth FM , Amorim AB , Parambath S , Shanmuganathan S , Huckel Schneider C . Implementation initiatives to improve low back pain care in Australia: a scoping review. Pain Med. 2022;23(12):1979–2009. 35758625 10.1093/pm/pnac102 PMC9714528

