# Supplementary material for: What is cancer pain? Investigating attitudes of patients, carers, and health professionals: A cross‐sectional survey
Source: Pain Pract. 2025 Mar 6;25(3):e70018. doi: 10.1111/papr.70018 (PMC11883519; doi:10.1111/papr.70018)
Supplement: Supplementary file 1 — Appendix S1. [file PAPR-25-0-s004.pdf]

Appendix 1: Preamble and implied consent for survey

Cancer pain experience in a Dedicated Cancer Centre

Part I: Implied consent screen

1. Introduction

You are invited to participate in a research project exploring cancer pain management understanding among health professionals and the community. This project aims to improve knowledge surrounding pain management strategies for individuals affected by cancer.

Participation is voluntary and all questions are optional. Responses cannot be identified and, once submitted, there will be no opportunity to withdraw your response.

The survey will take up to 10 minutes.

2. Are there any risks or potential benefits from participation in this evaluation?

You will have the opportunity to contribute potential insights for enhancing cancer pain management strategies, with the potential to improve overall patient outcomes and the experience of both staff and patients.

The participation in this study only involves reflecting on your personal views and experiences regarding cancer pain management. No performance or personal information will be sought from you. Therefore, we anticipate minimal risk of harm to you. Additionally, you have the option to discontinue the survey at any time. As survey responses are anonymous, you will not have the opportunity to withdraw after submitting your response.

3. What happens if I feel uncomfortable or distressed as part of the research?

During the survey, you can decline to answer any or all questions or ask to pause or stop participating at any time - without any explanation or consequence. However, in case you experience any distress or discomfort because of any part of this research, you can contact:

Lifeline (Ph: 13 11 14)

Beyond Blue (Ph: 1300 22 4636), 24-hour national counselling services.

4. What will happen to the information about me?

During the evaluation all survey data will be stored in a password protected electronic storage folder that can only be accessed by members of the research team. All information collected from the research, including your survey responses, will be managed and stored in accordance with the Privacy Act 1998.

Published research data will be kept for at least five years from the date of publication. After that time, digital information will be erased, and any hard copy documents will be securely disposed of.

5. How will the results of this evaluation be published?

The full report and detailed findings will be for the internal use of the Department of Anaesthetics, who may publish, or circulate to relevant stakeholders, a summary of the evaluation findings and recommendations (containing only aggregated, de-identified information). This may also include presenting summarised findings at scientific conferences.

6. What if I have questions about the evaluation?

If you have any questions about this evaluation, you may contact either:

Eamon Henriksen or Ciara Power: (03) 8559 5000

By pressing "Continue" you are consenting to data gathered from your answers to used as part of scientific research as stated above.

Continue
