# Supplementary material for: What is cancer pain? Investigating attitudes of patients, carers, and health professionals: A cross‐sectional survey
Source: Pain Pract. 2025 Mar 6;25(3):e70018. doi: 10.1111/papr.70018 (PMC11883519; doi:10.1111/papr.70018)
Supplement: Supplementary file 2 — Appendix S2. [file PAPR-25-0-s003.pdf]

Appendix 2: Demographic details form

Part II: Demographic details

1. **Age:**
   - ○ 18-30
   - ○ 31-50
   - ○ 51-70
   - ○ >70

   reset

2. **Sex:**
   - ○ Male
   - ○ Female
   - ○ Other (Please Specify):
   - ○ Prefer not to say

   reset

3. **Do you identify as Aboriginal or Torres Strait Islander**
   - ○ Yes
   - ○ No
   - ○ Prefer not to say

   reset

4. **Are you a health professional?**
   - ◉ Yes
   - ○ No

   reset

Please specify your clinician type:

- ○ Nurse
- ○ Medical Oncologist
- ○ Surgical Oncologist
- ○ Radiation Oncologist
- ○ Allied Health Clinician
- ○ Internal Medicine Clinician
- ○ Pain Specialist
- ○ Palliative Care Clinician
- ○ Administration
- ○ Other (please specify):

reset
