# Supplementary material for: What is cancer pain? Investigating attitudes of patients, carers, and health professionals: A cross‐sectional survey
Source: Pain Pract. 2025 Mar 6;25(3):e70018. doi: 10.1111/papr.70018 (PMC11883519; doi:10.1111/papr.70018)
Supplement: Supplementary file 3 — Appendix S3. [file PAPR-25-0-s001.pdf]

Appendix 3: Survey questions for health professionals

Part III: Survey Questions

Cancer pain survey for health professionals

	Agree	Unsure	Disagree	
Cancer pain is unique and different from other forms of non-cancer pain.	○	○	○	reset
Pain resulting from cancer treatment is also considered cancer pain.	○	○	○	reset
The only source of cancer pain is from the tumour itself.	○	○	○	reset
Managing cancer pain is complex.	○	○	○	reset
Non-pharmacological (physio, OT, mindfulness etc) pain management modalities can also alleviate cancer pain.	○	○	○	reset
A step ladder approach should be the basis of opioid analgesic escalation in management of cancer pain.	○	○	○	reset
The use of solely pharmacological analgesia is enough to achieve managed cancer pain.	○	○	○	reset
A multidisciplinary (medical and allied health) approach to cancer pain management is gold standard care.	○	○	○	reset
Pain specialists only manage non-cancer pain.	○	○	○	reset
It is difficult to refer to a pain specialist.	○	○	○	reset

Cancer is always associated with pain.	○	○	○	reset
The goal of pain management is to eliminate pain.	○	○	○	reset
Cancer pain can be properly assessed formulaically.	○	○	○	reset
Patients and carers need pain education to understand and manage cancer pain effectively.	○	○	○	reset
Cancer pain patients should have to abide by the same regulations in re to accessing opioids as non-cancer pain patients.	○	○	○	reset
Under the current regulatory systems (safe script, limited supplies, etc.), accessing pain relief is fair for non-palliative cancer patients.	○	○	○	reset
Strong opioid meds should be easily accessible for people experiencing cancer pain.	○	○	○	reset
Access to a pain specialist is a barrier to cancer pain management.	○	○	○	reset
Palliative Care teams play a central role in the comprehensive management of cancer pain.	○	○	○	reset
All cancer patients experiencing pain should be engaged with a Palliative Care service for pain management.	○	○	○	reset
There is a need for general education surrounding opioid medication in the community.	○	○	○	reset
Primary health professionals are hesitant in prescribing opioid analgesia.	○	○	○	reset
