# Supplementary material for: What is cancer pain? Investigating attitudes of patients, carers, and health professionals: A cross‐sectional survey
Source: Pain Pract. 2025 Mar 6;25(3):e70018. doi: 10.1111/papr.70018 (PMC11883519; doi:10.1111/papr.70018)
Supplement: Supplementary file 4 — Appendix S4. [file PAPR-25-0-s002.pdf]

Appendix 4: Survey questions for patients and carers

Part III: Survey Questions

	Agree	Unsure	Disagree	
Cancer pain feels different from other types of pain.	○	○	○	reset
Pain that arises from cancer treatment is considered cancer pain.	○	○	○	reset
Pain only manifests from the tumour itself.	○	○	○	reset
Pain specialists only manage non-cancer pain.	○	○	○	reset
Managing cancer pain is complex.	○	○	○	reset
Oncology teams are effective at managing cancer pain.	○	○	○	reset
Exercise, counselling and other non-medication-based methods can be used to manage cancer pain.	○	○	○	reset
Accessing tools to manage cancer pain (like medicine, counselling, and exercise) is an easy process for patients.	○	○	○	reset
Cancer pain can be treated with various approaches.	○	○	○	reset
Allied health should be included in the management of cancer pain (ie. physio, psych support, occupational therapy etc).	○	○	○	reset
